# A physical activity intervention to improve the quality of life of patients with a stoma: a feasibility study

**DOI:** 10.1186/s40814-020-0560-0

**Published:** 2020-02-05

**Authors:** Gill Hubbard, Claire Taylor, Angus J. M. Watson, Julie Munro, William Goodman, Rebecca J. Beeken

**Affiliations:** 1 Department of Nursing and Midwifery, Centre for Health Science, University of the Highlands and Islands, Old Perth Road, Inverness, IV2 3JH UK; 2 St Mark’s Hospital, London North West University Healthcare NHS Trust, Harrow, Middlesex HA1 3UJ UK; 3 Department of Surgery, Raigmore Hospital, NHS Highland, Old Perth Rd, Inverness, IV2 3UJ UK; 4 Leeds Institute of Health Sciences, University of Leeds, Worsley Building, Clarendon Way, Leeds, LS2 9NL UK

## Abstract

**Background:**

We hypothesise that a physical activity (PA) intervention will improve the quality of life (QoL) of people with a stoma. A feasibility study of the intervention and trial parameters is necessary to inform a future main trial.

**Methods:**

Participants received a weekly PA consultation by telephone, video conferencing, or face-to-face for 12 weeks with a PA instructor who prescribed physical activities and supported participants by addressing stoma-related concerns and using behaviour change techniques. A feasibility study of the intervention and trial parameters was conducted in three UK sites using mixed methods.

**Results:**

The number of eligible patients consenting to the study was 30 out of 174 (17%). Most participants were female (73%); 73% had an ileostomy and 27% a colostomy; mean time since diagnosis was 6 months. A total of 18 (64%) participants completed pre- (baseline) and post-intervention (follow-up) measures. Results show an improvement on all scales measuring QoL and disease-specific fatigue. The median PA consultation rate per participant was eight sessions. Participants reported completing 75% or more of the prescribed PA each week. Eight stoma-related themes were identified from qualitative interviews: fear of hernia, bending down, fatigue, pain, prolapse, surgical wounds, stoma appliance, and stigma. The intervention appeared to address these issues.

**Conclusion:**

This feasibility study demonstrated that a novel manualised PA intervention for people with a stoma is safe, feasible, and acceptable, and shows promise for improving outcomes. However, difficulties with recruitment will need to be carefully considered to ensure the success of future studies in this area.

**Trial registration:**

ISCTN, ISRCTN58613962; Registered 14/9/2017.

## Introduction

In Europe, approximately 700,000 people are living with a stoma, and in the USA, more than 1 million people have a stoma [1]. In the United Kingdom (UK), a national audit shows that just under 11,500 patients diagnosed with rectal cancer each year have a stoma formed [2] and a UK charity website indicates that each year, around 2000 people with inflammatory bowel disease (IBD) have a stoma formed [3]. A stoma is an artificial opening on the surface of the abdomen that has been surgically created in order to divert the flow of faeces or urine [4]. The two types of bowel stomas are colostomy and ileostomy, which can be temporary or permanent [4]. There are a number of conditions that may necessitate the formation of a stoma including bowel cancer and IBD [4]. Treatments and treatment-related side effects differ by disease with bowel cancer often treated by adjuvant chemotherapy and radiotherapy whereas anti-inflammatory drugs are often the first step in the treatment of IBD. IBD occurs mostly in young adulthood [5] in contrast with bowel cancer which occurs mostly in older age [6]. However, both patient groups will include some people that have a stoma formed as part of their treatment [2, 3].

Research shows that stomal complications include pain, prolapse, and parastomal hernia [7, 8]. The most common stomal complication is parastomal hernia [7, 9], which occurs when other abdominal contents protrude through the defect in the abdominal wall created for a stoma; prevalence is estimated to be over 30% by 12 months, 40% by 2 years, and 50% or higher at long duration of follow-up [10]. Studies highlight a trend toward inactivity after stoma formation surgery, with fear of hernia a major deterrent to being physically active [11–13].

Recent systematic reviews show that a stoma has a negative impact on quality of life (QoL) [14–16]. A comparative study of 331 patients with a permanent stoma compared to 117 patients without a stoma shows that fatigue is a greater problem in those with a stoma, and in the stoma group, a bulge or a hernia around the stoma and fear of leakage further impaired QoL [17]. Research about lived experiences and psychosocial health following stoma formation highlights three key themes: psychosocial impact around feeling of loss of control of body function, physical aspects that affect psychological function and QoL, and the process of acceptance, adaptation, and adjustment [18]. A recent patient survey suggests that just under half of patients with a stoma experience high levels of stigma [19].

Interventions are needed that have the potential to improve the QoL for this group of patients. Physical activity (PA) has been identified by patients with a stoma as a research priority in relation to their QoL [20]. No studies have been conducted about associations between PA and patient-reported outcomes in people with a stoma. However, there is some evidence about PA and QoL in patients who may have a stoma formed as part of treatment. A recent systematic review found that PA was positively associated with QoL in people ≥ 5 years post-diagnosis of bowel cancer [21], and emerging evidence suggests an association between PA and QoL in people with IBD [22]. Among people with a diagnosis of bowel cancer, those with a stoma are less likely to engage in PA than those without a stoma (odds ratio (OR) = 1.51, 95% confidence interval (CI) = 1.12–2.04) [21]. Furthermore, two recent surveys found that people with a stoma report a reduction in PA following stoma formation [11, 12]. Similarly, several barriers undertaking PA have been reported by people with IBD including abdominal/joint pain, fatigue/tiredness, disease flare-up, and increased toilet urgency [23].

Based on this body of work, we hypothesise that a PA intervention will improve the QoL of people with a stoma. Before embarking on a full randomised controlled trial to test this hypothesis, we developed a manualised PA intervention and assessed the feasibility and acceptability of implementing the intervention and study procedures for the future main trial. The objectives of this feasibility study were (1) to develop a PA intervention for people with a stoma, (2) to explore PA instructors’ experiences of delivering the PA intervention, (3) to assess the level of patient engagement with the PA intervention and their views on intervention acceptability and usefulness, and (4) to assess screening, eligibility, consent, data completion, loss to follow-up, missing data rates, representativeness of participants, and potential treatment effects. No pre-defined progression criteria were used to inform decision-making whether to proceed to a full main trial. Instead, the intention was to discuss the findings with the Patient Advisory Group and clinicians with a view to determining next steps including whether to conduct further feasibility and/or pilot work or proceed immediately to a full trial.

## Methods

A protocol of this study has been published [24]; hence, a brief summary of methods is provided here.

### Setting and eligibility criteria

Participants were recruited from three National Health Service Trusts/Boards: NHS Highland (Scotland), London North West Healthcare Trust, and University College London Hospitals NHS Foundation Trust (England).

People were eligible for inclusion if they were:

- Diagnosed with stages I–IV bowel cancer or diagnosed with IBD
- Greater than 6 weeks and < 24 months since stoma formation (permanent or temporary) surgery (laparoscopic or open surgical procedure)
- Willing and able to provide written informed consent

People were excluded if they had:

- Emergency surgery for stoma formation
- A clinician recommendation that they should not engage in any type of PA
- Ongoing adjuvant therapy (chemotherapy or radiotherapy)

### Assessing study procedures

Screening, eligible patients’ consent rate, the loss to follow-up rate, and reasons for excluding patients were recorded to assess recruitment procedures. Clinical and demographic information about participants were recorded to assess participant representativeness. Data completion and missing data rates for patient-reported outcomes were recorded to assess the acceptability of the proposed outcomes for a future trial. All serious adverse events (SAE) and adverse events (AE) were recorded regardless of considered link with intervention or study participation.

### Assessing outcomes

Stoma-related QoL was measured using the Stoma-QoL [25]; bowel cancer-related QoL (bowel cancer patients only) was measured using the Functional Assessment of Cancer Therapy (FACT-C) [26]; IBD-related QoL (IBD patients only) was measured using the Short Inflammatory Bowel Disease Questionnaire (SIBDQ) [27]; fatigue was measured using the FACIT Fatigue Scale [28]; physical activity was measured using the Actigraph GT3X+ accelerometer (Actigraph LLC, Pensacola, FL, USA) [29]; systemic inflammation (IBD patients only) was measured through biomarker analysis of blood samples.

### Intervention

A manualised PA intervention was developed by the research team (which also included clinicians), Patient Advisory Group, and PA instructors who met on two separate occasions. Their main respective contributions included the following:

*Researchers—*theories of behaviour change and behaviour change techniques. The clinicians on the research team also provided information about bowel disease and stoma.

*Patients—*lived experience of stoma and being physically active (or not).

*PA instructors—*practical advice about exercise.

The produced Manual is available on the Bowel & Cancer Research website (https://www.bowelcancerresearch.org/Handlers/Download.ashx?IDMF=c3d26c0d-c746-4c96-89dd-d66124d2721e) and includes sections about bowel diseases and stoma, PA guidelines, PA consultations, PA prescriptions, goal-setting, PA preferences, PA benefits, motivating people to engage in PA, behaviour change techniques, pedometers, PA diaries, precautions for PA in patients with bowel disease, precautions for stoma hernia, solutions to address concerns about being active, experiences of being active, useful tips and resources, and frequently asked questions.

The intervention that we developed and tested is briefly described below using the Template for Intervention Description and Replication (TIDierR) subheadings and guidance [30]:

*Why—theory and components*. We hypothesised that a PA intervention would improve QoL. The intervention is based on self-determination theory [31] which focuses on maintaining the motivation to be physically active by making sure that participants have ‘autonomy’, are ‘competent’, and experience ‘relatedness’ [32]. Behaviour change techniques relating to these constructs were highlighted in the Manual for instructors to use, including use of pedometers to facilitate self-monitoring of outcomes of behaviour [33].

*What—materials*. A PA instructor used the PA intervention Manual to guide how they supported participants in the study. Participants were given a pedometer to self-monitor their step count each day for 12 weeks. Participants were also given a diary to record their daily step count and the weekly physical activities prescribed by the instructor. Participants recorded in the diary if they managed to complete the prescribed PA each week for 12 weeks using a continuous rating scale: all of it (100%), most of it (75%), some of it (25%), and none of it (0%).

*What*—*procedures.* Participants were expected to have 12 (1 per week) consultations with an exercise instructor. The first consultation was face-to-face followed by 11 telephone, video conferencing, or face-to-face consultations.

*Who provides, how and where*. A PA instructor delivered the intervention in each of the three research sites. Instructors were all qualified to Register of Exercise Professionals (REPs) Level 4 in Cancer and Exercise (www.canrehab. co.uk), and they attended two education sessions with the research team and members of the Patient Advisory Group about the Manual, stoma care, and barriers to PA for people with a stoma. Hence, the instructors received identical education in order to deliver the intervention. Participants received a weekly PA consultation from an instructor in-person, telephone, and by video conferencing depending on participant preference and internet facilities at home.

*When and how much*. The intervention was of 12 weeks duration. Each participant received a weekly consultation that was expected to last between 30 and 60 min. The instructor prescribed physical activities for the participant to complete each week. A typical prescription was expected to be 30 min of aerobic activities, 2 sets of 12 repetitions for muscular strength and endurance, and flexibility and balance activities. However, weekly prescriptions were expected to vary from one participant to the next.

### Assessing intervention implementation

*Intervention fidelity* was measured by the total number of consultations delivered by the instructor and explored through individual semi-structured face-to-face or telephone interviews (depending on participant preference) with participants and instructors. *Intervention adherence* was measured by participants’ completion rate of the physical activities prescribed by the instructor. *Intervention acceptability* was explored through individual semi-structured face-to-face or telephone interviews with participants.

### Sample size

It is inappropriate to base feasibility study sample sizes on measures of intervention effect, which is the purpose of the full-scale trial. We aimed to recruit 30 participants, which we believed would provide a sample of participants with different demographic and clinical characteristics that would improve our confidence in the conclusions we drew from the feasibility study [34].

### Recruitment

Three recruitment methods were used:

*Prospective—*a stoma/colorectal nurse specialist discussed the study face-to-face with patients that they had screened previously for eligibility on the ward or at an out-patient clinic.

*Retrospective—*the clinical team sent a letter of invitation to patients that they had screened previously for eligibility (all three sites used this method).

*Social Media—*an advertisement about the study was disseminated by members of the Patient Advisory Group and by relevant stoma charities on both Facebook and Twitter.

For all three methods of recruitment, all potential participants either contacted a researcher directly or consented to be contacted by a researcher. Then, a face-to-face meeting was arranged where a researcher explained the study in further detail and took informed consent in writing.

### Data collection

To collect data to assess *intervention implementation*, the instructors kept a paper record of each consultation and participants completed a weekly diary. Instructors and participants were interviewed by a researcher at the end of the study. To collect data to assess *study procedures*, researchers kept a record of recruitment and data completion rates. *Outcomes* were measured at baseline and immediately after the 12-week intervention using the following procedure: each participant met with a researcher face-to-face to complete questionnaires (QoL and fatigue measures) hosted by Bristol Online Survey. Participants were given an accelerometer to wear for 7 consecutive days. Participants with IBD only had bloods taken by a nurse, and the researcher sent the sample to the hospital laboratory for testing.

### Data analysis

#### Descriptive statistics

Screening, eligibility, consent and data completion and missing data rates, and reasons for excluding patients were summed and reported as percentages. The consultation and completion of prescribed physical activities rates were summed and reported as percentages. Averages for step count and consultation duration (in minutes) were calculated. The estimated effect on outcomes were calculated and presented by the mean difference between baseline and follow-up, with the associated 95% confidence interval following paired *t* test analysis. This is because the focus of the results from this feasibility study will be on the estimates of treatment effects rather than statistical significance and hypothesis testing, which is the purpose of a full trial. The number of adverse events was summed.

#### Analysis of interview dataset

A process of induction was initially followed to allow for codes to emerge direct from the interview dataset. Induction is a process that can code the data without trying to fit it into any pre-existing coding frame or theoretical model [35]. To structure the data inductively, an initial set of codes were set by identifying recurring words, for example, hernia, fatigue, routine, instructor, pedometer, and enjoyment. These codes denote what words kept reoccurring throughout the dataset. Coding of data showed that participants predominantly spoke about their experiences of barriers and facilitators to PA and how acceptable they perceived that the PA intervention addressed these barriers. Instructors spoke about how they supported participants to address these barriers and use of behaviour change techniques, which was important qualitative data for our interpretation of intervention fidelity. During the second stage of analysis, we drew on an ecological model of the determinants of physical activity, which highlights individual, interpersonal, and environmental barriers and facilitators of PA. In stage two, coding was re-visited and data were categorised by theme under each determinant of PA. All themes were divided into one of two categories: (1) related to stoma or (2) not related to stoma.

### Patient advisory group

A Patient Advisory Group was set up primarily for the following reasons: (i) advise on the design of the study to support the initial application for research funding, (ii) develop the PA intervention Manual and ensure it was delivered safely and to the benefit of study participants, (iii) assist with recruitment, and (iv) disseminate the study findings to relevant groups and individuals.

Members were recruited from previous studies conducted by the research team and by approaching relevant charities. The PAG grew from an initial membership of 7 to its current membership of 16. No members were paid; travel and subsistence expenses were covered by the research grant.

Communication between the research team and the Patient Advisory Group was predominantly by email if the purpose was to seek feedback on information sheets, consent forms, etc. Four face-to-face meetings took place for other purposes such as developing the intervention Manual, producing a brief film about living with a stoma and PA, and delivering training to the three PA instructors about stoma and PA.

## Results

### Study procedures (Fig. 1)

Recruitment

Screening data (i.e. a complete patient list with eligible and ineligible patients identified) was recorded in two sites that used the retrospective and prospective methods of recruitment. Screening was not applicable for the social media method of recruitment. Two hundred and fifty-eight patients were screened in the two sites, and of these, 120 (47%) were eligible. Reasons for ineligibility were available in 2 sites for 61 out of the 138 patients who were screened and deemed ineligible. Reasons were emergency surgery, *n* = 29; active chemotherapy, *n* = 14; pending or completed stoma reversal, *n* = 16; and deceased, *n* = 2. One site did not provide these data due to failure on the part of the research team to establish processes to systematically and consistently provide these data.

**Fig. 1:**
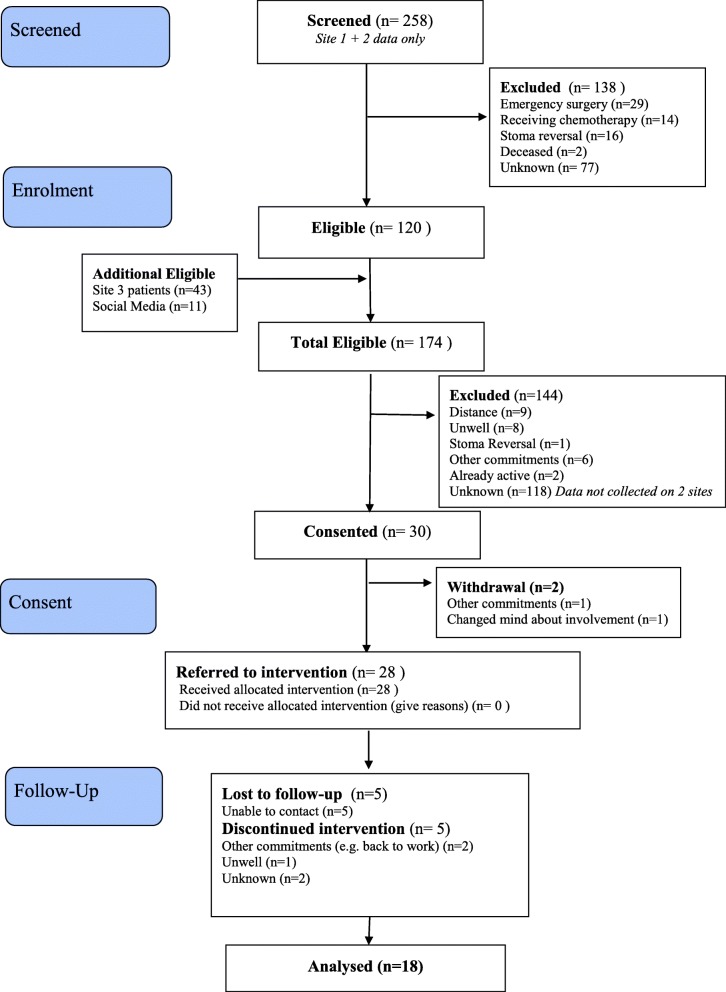
Participant flowchart

The total number of eligible patients across all three sites using the retrospective and prospective methods of recruitment and via social media was 174. The total number of eligible patients consenting to the study was 30 out of 174 (17%). Consent rates varied by site: 10 out of 43 (23%), 3 out of 51 (5%), and 7 out of 69 (10%), respectively. Consent rates also varied by method of recruitment: 10 out of 34 eligible patients (29%) consented by the *prospective* method of recruitment, 12 out of 129 (9%) consented by the *retrospective* method of recruitment, and 8 out of 11 (72%) consented by the *social media* method of recruitment, respectively. Reasons for not consenting were available in 1 site for 26 out of 51 eligible patients. Reasons were distance to meet instructor, *n* = 9; patient felt too unwell, *n* = 8; stoma was due to be reversed, *n* = 1; other commitments (back to work, family commitments), *n* = 6; and already back to normal activity levels, *n* = 2.

#### Participant characteristics

The clinical and demographic characteristics of consenting participants are presented in Table 1. Most participants were female (73%); mean age was 52 years; 44% were diagnosed with colorectal cancer and 53% with IBD; 73% had an ileostomy and 27% a colostomy. The mean time since diagnosis was 6 months (minimum 1 month; maximum 20 months).

**Table 1:** Sample characteristics (*n =* 30)

Variable		*n*
Gender	Male	8 (27%)
	Female	22 (73%)
Age (years)	All	Mean 52 (min 24; max 77)
	Colorectal cancer only	Mean 63
	IBD only	Mean 46
Diagnosis	Bowel cancer	11 (37%)
	Rectal cancer	2 (7%)
	Crohn’s disease	7 (23%)
	Ulcerative colitis	9 (30%)
	Unknown	1 (3%)
Type of stoma	Colostomy	8 (27%)
	- Colorectal cancer	7
	- IBD	1
	Ileostomy	22 (73%)
	- Colorectal cancer	6
	- IBD	16
Time (months) diagnosis		Mean 6 (min 1; max 20)

#### Loss to follow-up and missing data

Two out of the 30 participants dropped out of the study before baseline measures. Twenty-eight participants completed baseline measures, and 18 (64%) of these completed follow-up measures. There was an association between finishing the 12-week PA intervention and completing follow-up measures. Twenty-three participants completed the full 12-week intervention, and of these, 18 (78%) completed follow up-measures compared to 5 who did not complete the intervention nor complete follow-up measures.

The missing data rate for the 18 participants who completed baseline and follow-up measures for each outcome measure was Stoma-QoL *n* = 56% (missing questions included work *n* = 28% and sexuality *n* = 44%), FACT-C *n* = 10%, SIBDQ *n* = 0%, FACIT *n* = 0%, and objectively measured PA *n* = 0%.

#### Inflammation

Inflammation as a trial outcome required participant blood samples to be taken. This process required an NHS ethical committee amendment. The time taken to obtain NHS ethical and local Research Management approvals for the amendment meant that it was not possible to systematically collect blood samples. Only two participants had blood samples taken in one site, and the decision was made to cease any further blood sample collection and not to proceed with this analysis.

### Outcomes

Estimated treatment effects are as follows: Paired samples *t* tests were conducted to assess the mean change in stoma and disease-specific QoL scales and fatigue (Table 2). Results show an improvement on all scales measuring QoL and disease-specific fatigue; the greatest increases were observed in the FACT-Colorectal scale (28.1; 95% CI 15.8, 40.4) and the skin irritation (20.6; 95% CI 2.8, 38.3) and work/social function (15.7; 95% CI 7.2, 24.2) subscales of the stoma-related QoL. Minimal important differences (MID) were observed for FACT-Colorectal (increase of 28.1, MID = 5–8) and FACIT-Fatigue (increase of 5.0, MID = 2.8–6.8) outcomes.

**Table 2:** Results of the paired *t* tests showing mean difference between baseline and follow-up

Scales (range)	*N*	Baseline	Follow-up	Mean difference	95% CI
Stoma-related QoL (0–100)^a^	8	65.9	74.3	8.4	3.2, 13.6
Work/social function subscale	13	54.2	69.9	15.7	7.2, 24.2
Sexuality/body image subscale	10	66.0	75.0	9.0	− 0.4, 18.4
Stoma function subscale	16	64.8	65.1	0.3	− 7.2, 7.8
Financial concerns subscale	14	89.3	96.4	7.1	− 9.3, 23.6
Skin irritation subscale	17	41.2	61.8	20.6	2.8, 38.3
FACT-Colorectal (0–136)^a^	9	78.3	106.5	28.1	15.8, 40.4
SIBDQ QoL (10–70)^a^	8	47.1	53.5	6.4	− 1.9, 14.7
FACIT-Fatigue (0–52)^b^	18	33.9	38.9	5.0	0.9, 9.1
Objectively measured physical activity (step count)	17	7542	8401	858	− 3500, 1792
Objectively measured physical activity (minutes moderate to vigorous activity (MVPA))	17	73	81	8	− 30, 14

FACT-Colorectal minimal important difference 5–8 [36]. FACIT-Fatigue minimal important difference 2.8–6.8 [37]. *QoL* quality of life, *SIBDQ* Short Inflammatory Bowel Disease Questionnaire

^a^Higher scores indicate higher QoL

^b^Higher scores indicate less fatigue

No adverse events were reported.

### Intervention implementation

Thirty participants were recruited to the study; hence, the total maximum number of consultations that all participants could potentially receive was 360 (30 participants × 12 weeks). Two participants withdrew from the study. A total of 206 consultations were conducted (57%). The median consultation rate per participant was 8 sessions. Of 206 consultations, 97 (47%) were face-to-face, 18 (9%) by video conferencing, 18 (9%) via email discussion, and 73 (35%) by telephone. The duration of the consultation ranged from 15 to 45 min (minimum 5 min, maximum 120 min, median 35 min). Twenty-two out of 28 physical activity diaries were returned. Two diaries were incomplete and removed from analysis. Of those that did return a completed diary (*n* = 20), the average number of weeks that participants completed the prescribed activity was 10 (out of 12) (80%). Completion was reported by participants as 75% or more of the prescribed activity for each week. Six minor stoma issues were reported in the diary entries; only two of these issues were related to PA (1 abdominal discomfort on bending; 1 feel of pulling on bag adhesive when stretching). The mean daily step count, which was used so that participants could monitor PA (a common behaviour technique) was 8175 (range 187–35,656). The missing data rate for daily step count was 4% (69/1665 entries missing).

### Qualitative interviews

Fourteen interviews were conducted with 14 participants and 3 instructors. Interviews were conducted at a time and place convenient to participants and instructors. Thirteen were face-to-face, and 4 were by telephone. Themes are presented in Table 3.

**Table 3:** Themes from qualitative interviews

Determinants of PA	Themes related to stoma	Themes not related to stoma
Individual	Fear of hernia Bending down Fatigue Pain Prolapse Surgical wounds Stoma appliance	General fitness Emotions (frustrated, upset, bored) Routine (BCT) Monitoring (BCT) Goal-setting (BCT) Injury Preferences for type of PA (BCT) Determination to be active Ageing Dog walking
Interpersonal	–	Social support—instructor (BCT) Social support—family (BCT) Social support—friends (BCT)
Environmental	Stigma	Shopping Travel Work Holidays Weather Money

*BCT* behaviour change techniques

Only themes that related to stoma are reported with quotations from participants below. Other themes such as ‘routine’ and ‘monitoring’, ‘goal-setting’, ‘preferences’, and ‘social support’ have been consistently highlighted in the literature as important determinants of PA and hence are not repeated in this paper [33, 38]. Routine, for instance, is a common behaviour change technique, which helps participants make PA habitual [33]. There was nothing in participants’ accounts to suggest that ‘routine’ was related to stoma. Another example of a behaviour change technique that was not related to stoma is ‘monitoring’ [33]. Each participant was given a pedometer to wear in order to encourage use of the behaviour change technique, ‘self-monitoring of behaviour’ [33]. Again, there was nothing in participants’ accounts to suggest that monitoring was related to stoma. Moreover, most of the environmental determinants of physical activity were not stoma-related, which was a point highlighted by one of the PA instructors:

Yes. Barriers? Just the same as everybody else, so time, money, location, prioritising exercise.

Instructor A: 61

In the ‘Results’ section that follows, we report the eight themes directly relevant to stoma because these are novel findings. Stoma-related themes are fear of hernia, bending down, fatigue, pain, prolapse, surgical wounds, stoma appliance, and stigma. Quotations to illustrate themes were chosen by the researchers and have a unique participant identifier and line number for referencing purposes. We highlight if several participants’ data is associated with a theme or if it is only one participant. This is not to imply a hierarchy of importance but rather to give a sense of what issues were more common than others in this study.

#### Fear of hernia

Fear of increasing the risk of hernia was a key barrier to PA that the intervention appeared to address. One participant said that the advice and guidance she received following stoma formation surgery made her cautious about being physically active.

Participant: But when you’re getting all the information and all the emphasis on, “Watch you don’t get a hernia. Careful of a hernia.” And you’re thinking, “Well, if I do this, will I get a hernia? Will I strain myself?”

Researcher: Yeah. Was that a fear?

Participant: It was a bit, yeah. Because when my stoma was badly swollen, that’s what you’re thinking, “Oh, have I got a hernia?” But then I had an appointment with the stoma nurse in January, February? February, I think it was. And I’d said to her, and she checked me out for a hernia. So, that put my mind at rest.

Researcher: So, who was advising you to be careful of a hernia? Where did that advice come from?

Participant: That came from the stoma nurses and all the information packs. INV08:153-164

Instructors reported that many participants were unsure about how much and what types of activities they should be doing. Instructors noted that there was lack of information for people with a stoma about PA. One instructor said that lack of information made people unclear what they could do.

It was more a case of them not knowing, rather than being nervous about trying things. So, I think they maybe didn’t have enough information provided to them, maybe post-surgery, about what they can and can’t do. Yeah, but the main concern was people not being sure if they should be lifting, lifting things. So, and then obviously, you know, if you’ve got a strenuous job or something, and they were concerned about that. Instructor A: 46-50

Several participants said that they were scared of being physically active because it might increase the risk of hernia. Doing an exercise therefore required assurance from the instructor that the exercise was safe and would not increase the risk of herniation. One participant described how the instructor addressed her fear of hernia and gave her confidence to be active.

If I hadn’t had [name of instructor] to know it was okay to do various things I would have probably been reluctant because, you know, you’ve had your muscles cut and you might get a hernia and all that sort of stuff, and then you’ve got a bag that’s sticking out when you’re trying to fold your body in half, you know. So, all those things would… she sort of, not gave permission, but she gave you confidence that it was okay to do all those things. SOC02:101-106

Instructors played an important role in encouraging people to believe that it was safe to be physically active with a stoma. One participant described how the instructor put her at ease by talking to her about a hernia, being with her at the gym while she exercised, and encouraging her to exercise within her own limits.

And I’ve heard so much about hernias and everything, that was the biggie, I do not want one of them. So, after speaking to him [instructor], and then actually doing it in the gym with him, it sort of put you at ease. And then you knew what you can… as he said [instructor], you know your own limits. There’s obviously things that I can’t do which other people can do, but, yeah, it felt a lot more at ease about being able to do things and not have to constantly worry if I’m going to get a hernia, if I’m going to hurt myself, or if it’s going to affect my stoma. INV09:44-51

Showing participants how to perform an exercise was therefore a crucial part of the intervention. Hence, one of the instructors believed that the face-to-face consultation was important because it enabled them to show participants how to perform an exercise.

I thought the telephone consultations were fine. But I found it really, really important to do, especially when they came in for the first appointment when we did their functional assessment, but we also, I also prescribed and coached the exercises that I wanted them to do, I found it was very, very important to spend enough time, maybe more than enough time, just to reiterate and repeat techniques and, you know, the exercises that I was getting them to do, because they going to be left for a couple of weeks and they’re going to be exercising on their own, and what we don’t want is for them to be executing the exercises incorrectly and maybe picking up injuries, or just not exercising correctly. So, I really spent a lot of time with them in that first session, you know, going through, you know, the exercise program and the exercises that I want them to perform. Instructor R:121-131

One participant said that his biggest fear since having a stoma was hernia.

Yeah. I do worry about getting a hernia, like that is my biggest… my biggest fear about having it. Like, I’m not bothered about having the stoma whatsoever, it saved my life, but my biggest fear is having, getting a hernia and having to have that removed and, you know, having to deal with that. INV14:130-33

However, he felt re-assured that the instructor knew what he was doing and therefore was able to engage in exercise in the presence of the instructor.

Yeah, it was fine. It’s a little bit scary because, of course, everywhere that you look, people are going on about hernias and, you know, all this kind of thing. But I think if you’ve got somebody there that actually knows what they’re doing, it’s fine. INV14:40-45

A technique that instructors used to address fear of hernia was by progressing an exercise so that participants became more confident in their technique and ability to perform an exercise safely.

Instructor: Yeah, that kind of conversation a few times, is the fear of, I think a few of them had hernias in the past, and they were concerned about getting another one. But, no, we just went through good kind of manual handling technique, I suppose, thing like deadlifts. Varying exercise, so, typically, you know, I’d have someone deadlifting a bar, so, but for some of them, it was a kettlebell that was raised up on a platform. So, to make the easier for them to build their confidence up.

Researcher**:** So, you’re adapting things?

Instructor: Yeah. And it’s just a case of progressing the intensity as they increase their confidence. But, yeah, once you get them going and they know they can do it, then that’s the main thing.

Instructor A: 159-168

Some participants highlighted that the PA intervention had addressed their fear of hernia and dispelled their perception that exercising increases hernia risk. One participant no longer believed that exercise increased the risk of hernia.

Participant: Yeah, mainly because I would be scared of what I could do, because that’s probably the worst thing about Facebook groups, because I’m in Facebook groups like for… and that’s all you see, is people saying about hernias and things… Just because it just seems that a lot of people on the Facebook group have hernias. And the stoma nurse and even [name of surgeon] said, “You don’t have to panic, you’re not a high risk for one. And if it happens, it happens, we’ll sort it.” But still you’ve got that, and you’re just scared that maybe if you’re doing weights and anything, if you lift the wrong way, and that occurs.

Researcher: So, what’s your feelings on that now?

Participant: Doesn’t bother me. I don’t think physical exercise is the reason why you’re going to get a hernia. INV09:69-84

As far as the instructors were concerned, there was nothing that someone with a stoma was notable to do as long as they worked to their own abilities and fitness levels.

No, but I think the main thing to reiterate is, in terms of exercise selection, there wasn’t anything I stayed clear of in terms of, you know, being too, too wary of trying certain things. It was just a, just normal sessions, and just going with people’s own abilities and fitness levels. Instructor A: 259-262

#### Bending down

Several participants reported that the stoma had made bending down difficult. Difficulty in bending down meant that some daily activities were harder to achieve than others. Participants reported that the PA intervention had led to significant improvements in their ability to bend, which meant that they could perform daily activities that they had found almost impossible to do previously.

But I had a lot of issues with, like, bending down, specifically on my stoma side, I was really nervous about that and I was kind of hurting myself a bit in doing that… But I feel like it has definitely given me more grounding in my body. I’m not scared of it, I think I’m just more aware of, like, like when I started with the program I was having trouble putting on my sock on my stoma side because I was having trouble bending down, and I was like “this is my life forever now”, and now it’s not a problem, I have much more flexibility, I’m realising that, like, I can have these things, I just need to kind of work at it. UCL39:17-92

I couldn’t paint my own toenails, when I can now. Well, you know, that’s quite a benefit, that one can bend down a bit further than I could. UCL26:73-73

I now am able to almost touch my toes when I’m sitting down. HAR04:13

#### Fatigue

Several participants said that fatigue had a negative effect on PA, which was expressed as feeling tired, exhausted, and lacking in energy. Participants described fatigue as almost impossible to overcome, but several had found the intervention helped.

One participant referred to her fatigue as chronic and unpredictable but reported that the intervention had made her feel physically so much better.

The only problem is that I have fatigue. So, my, and it’s a real chronic fatigue now. And that is my frustrating problem because it doesn’t, you’re sort of on one level, and then you dive and there’s nothing to say that you’re going and tiring, you just suddenly go. And that is, that’s the only thing that’s holding me back. But physically, my muscles are better, my mobility, everything is better. HAR04 line 120-124

Several participants said that the PA intervention had helped to counteract the problem of fatigue.

Participant: They [people who also had a stoma formed surgery] couldn’t believe I was back at work, They couldn’t believe I was in the gym, they couldn’t believe I was on the treadmill every night…they all saying that they’re tired all the time, and I’m just now. And, you know, I have said, ‘You should maybe think about doing something, you know, even if it’s just walking a bit more during the day.’

Participant: Do you think it’s [the physical activity intervention] given you more energy?

Participant: Yeah, 100%. Yeah. INV09 217-233

When asked if the PA intervention had been beneficial, one participant described how it had helped her combat exhaustion.

I mean, when I came here [to see the instructor a consultation] that first time, I was absolutely exhausted when I got back. In fact, coming through it was the first time I’d been on the Tube. So, when I got home it was like I couldn’t move, absolutely couldn’t move. But now I’m always on the Tube, so not a problem. Also, my recovery rate is quicker. So, where I would be absolutely exhausted and just have to lay on the bed and that was it for the rest of the day, now if I just sit and relax for ten, fifteen minutes, I’m just as good as what I was before. HAR04:126-130

#### Pain

Pain was another physical factor impeding PA. At the same time, the PA intervention was perceived by some participants as an antidote to pain.

I was getting quite a lot of pain… That’s the thing, the pain’s gone, not completely, but it’s gone quite. INV04:30-72

Some participants experienced pain surrounding the stoma during PA. One participant described how she and the instructor worked out together why she was experiencing pain and altered the equipment accordingly.

There was one week that I was, my stoma was actually really quite painful, but we realised it was the movement of the bike, because he had me on the small bike, and of course the movement like that legs hitting off, so all he did was put me on a proper bike, and I was fine. INV14:33-36

One instructor believed that participants were frightened and unsure what sensations around the stoma were normal.

I think some people were very frightened about what movements were safe and what to do. And any kind of twinge or kind of unusual sensation that they were getting around their abdomen, that made them a bit nervous. But, again, it’s just talking people through that dialogue that actually it’s quite normal to feel those sensations and perhaps the odd twinge and the odd pull as long as it’s obviously not painful. So, again, hopefully those things, those fears, were kind of laid to rest, really, once the…. Because often it is just that someone to say “actually, that’s quite normal”, but if you haven’t got anyone there to say “yeah, that’s okay as long as it’s not an unusual pain, it’s okay to feel that.” So, that’s sometimes a bit of a barrier, but I felt that we kind of worked through some of those. Instructor L:131-140

This instructor therefore believed that one of the most important roles that she had was making participants believe that it was safe for them to be physically active and encouraging them to be safe.

It was just giving people that reassurance that they could either return to what they were doing before, or springboard them into starting something new. Instructor L:10-12

#### Prolapse

Only one participant referred to the problem of prolapse. She said that she was protecting her stoma and reducing the risk of prolapse by not exercising. She believed that the PA intervention had helped her to reduce the risk of prolapse by helping her to de-stress.

Participant: And I think because I had kind of been protecting it [stoma] without realising it, I hadn’t consciously moved that area of my body in a long time... I had a very minor prolapse before the program started, and so sometimes my stoma is more likely to sort of not quite prolapse but sort of, and I’m terrified of that…

Researcher: And do you think the exercise helped with that at all?

Participant: Definitely. You mean that in terms of the prolapse, or?

Researcher: The prolapse or any other kind of stoma-related issues.

Participant: I think it does. Like, again, like I said, I haven’t done anything [exercise] for probably realistically close to two weeks now, because I’m going through a really intense time at university, and I am having more actually of those very similar issues right now. And so, I do think not exercising is partially contributing to that. I have a feeling it might also be a stress thing. UCL39:60-74

#### Surgical wounds

Only one participant referred specifically to surgical wounds. She was careful jogging because of her surgical wounds and managed this by incremental increases in how often she jogged.

And I’ve even, and this is a bit of a shock to me as well, done some jogging, which I have not done in a very, very long time. But I have to be careful with that one because, because of the surgical wounds and everything. It was a bit of a shock to the body, so I’m actually doing that very; it was probably jog for twenty seconds and then jog for, but I’ve got to a hundred and fifty metres. It’s still not a great deal, but for me it’s like amazing. HAR04:64-68

#### Stoma appliance

Only one participant referred to the stoma appliance. He said that the intervention had made him realise that being active was not the cause of appliance problems. Thus, similar to misconceptions about PA and hernia, the PA intervention re-educated people about PA and stoma appliances.

Confidence of actually doing exercises and knowing that my stoma is not going to, one of the pipes isn’t going to blow off. You know, silly little things like that, because that’s everything that goes through your head. That’s the things that you worry about. What if I have a leak in the middle of the gym? And, you know, stuff like that. So, I think knowing that that’s not going to happen, and if it does, it’s not because you’re in the gym. INV14:60-64

One of the instructors commented on the impact of exercise on the stoma appliance. When the researcher asked if there were any issues with the stoma that arose, he replied that the sweating affected the appliance. He also mentioned pain that was related to the medical condition rather than the appliance.

There was… no, I think there was only one issue, small minor issue, was I think sweating… when they were sweating obviously through physical activity, it was affecting like the stickiness of the… you know, where it would be. I think that was the only issue. Another patient had a few kind of pains but it wasn’t necessarily directly linked to the stoma. I think it was more a case of under investigation they found out that it was more her Crohn’s disease. Instructor R:83-88

#### Stigma

One environmental factor was associated with PA and having a stoma, which was stigma. Only one participant referred to stigma. She believed that being physically active would encourage other people with a stoma to be physically active and address stigma associated with a stoma, which was one of the main reasons why she consented to participate in the study. She believed that being active would challenge clinician misconceptions about stoma and PA, thereby highlighted a potentially wider impact of the PA intervention.

I wasn’t aware of this, but apparently there is a stigma around having a stoma, and that really upsets me… I think as a very confident young lady it’s important to show that you can still do things with stomas… And for me, actually the stoma wasn’t a stigma, it wasn’t a negative thing, it’s one of the best things that’s happened… And I just wanted to show that the stoma nurses, who I trusted when I had the surgery and said, “Will I run again?” and they said, “Hmm, we don’t think you will.” I wanted to show that actually because you’re a qualified medical professional doesn’t mean, and I’m not saying they’re wrong, but I’m just saying that actually you can do things with a stoma. It’s just education and being careful of the facts. So, I suppose I’m trying to be a helpful guinea pig to encourage others into an area that is often discouraged after surgery. SOC01:1-19

## Discussion

This feasibility study demonstrated that a novel manualised PA intervention for people with a stoma is safe, feasible, and acceptable, and shows promise for improving outcomes. However, difficulties with recruitment and completion of some outcome measures for instance need to be carefully considered to ensure the success of future studies in this area.

### Study procedures

A key purpose of a feasibility study is to estimate important parameters that are needed to design the main trial so that the main trial is internally and externally valid [39]. Hence, one of this feasibility study’s main objectives was to assess screening, eligibility, consent, data completion, loss to follow-up, missing data, and adverse event rates.

Recruitment data are important for assessing the extent to which a study is generalisable (external validity). Based on this feasibility study, we estimate that the future main study would have an eligibility rate of around 50%, which means that the findings would be generalizable to half of people who have bowel stoma formation surgery in the UK. To increase this rate would require changing the inclusion and exclusion criteria. One of the main reasons why patients in the study were ineligible was because of ‘pending or completed stoma reversal’. It is not appropriate in the future main study to change this criterion because the PA intervention purposefully targets people with a stoma. Another reason why patients were ineligible was because they were on ‘active chemotherapy’. Removing this criterion so that patients on active treatment could participate could potentially increase the eligibility rate; however, it is unlikely to increase the consent rate because one of the main reasons why eligible patients did not consent to the study was ‘feeling unwell’ and side effects of chemotherapy include feeling sick, tiredness, sore mouth, and diarrhoea [40].

Nonetheless, this feasibility study does highlight ways in which the consent rate could be improved in the main full trial. Using social media such as advertising the study on relevant Facebook groups (often closed groups), twitter feeds, and charity websites did increase the number of people in the study. The study varied by research site and method of recruitment, suggesting that there are potentially modifiable contextual factors influencing the consent rate. The prospective method, i.e. a direct face-to-face approach by a clinician, was a more successful method of recruitment than the retrospective method, i.e. a letter being sent by the clinical team. However, the prospective method relies on a commitment from clinicians to engage in recruitment, and as the literature highlights, there are a range of reasons why clinicians may not recruit to a study including their perceptions of the importance of the research question and clinical workload [41–43]. A future study should therefore ensure that each site, and in particular, those tasked with recruiting patients, has an interest and capacity to engage in the study in order to optimise the reach of the intervention.

The qualitative findings of this study highlight a key reason why the consent rate was low in this patient group, which is that many participants had a fear of hernia, and associated PA with increasing the risk of developing a parastomal hernia. Participants reported receiving conflicting information about the risks of engaging in PA. Cross-sectional surveys of people with a stoma have also highlighted fear of hernia as a barrier to PA [11–13]. Convincing clinicians and patients that PA is safe and beneficial will be important in the future main trial in order to maximise recruitment and therefore generalisability. This feasibility study highlights other reasons why eligible patients did not consent, such as other commitments (e.g. family and work). These reasons are common in PA intervention studies in people with bowel cancer [41] and cancer trial participation in general [44] and therefore likely to also feature in the future main trial.

Loss of participants during a trial’s follow-up can introduce bias and reduce statistical power, thereby affecting the validity and reliability of results [45]. An estimated 20% loss can threaten trial validity [46]. Some missing data can be dealt with statistically and therefore may be regarded as less of a problem than poor completion; nevertheless, the risk of bias and imprecision due to missing data can remain [47] and therefore should be reported alongside other rates. Given this is the first PA intervention study in people with a stoma, it is not possible to compare drop out, completion, and missing data rates with other equivalent studies. Nevertheless, drop out was low (only two participants) and the completion rate (64%) is similar to a recent UK feasibility study of a PA intervention in people with bowel cancer [48] and lower than home-based PA intervention studies in people with colorectal cancer carried out in other countries [49, 50]. This feasibility study does highlight one of the ways in which the completion rate could potentially be improved in the main future trial; those completing the 12-week intervention were more likely to complete follow-up measures. Hence, every effort should be made to ensure that participants engage in the intervention from start to finish (see next section for a discussion of intervention implementation). Missing data can be explained primarily by a failure to answer QoL questions about work and sexuality, which arguably are not relevant to all participants (some participants were not in work) or are perceived as sensitive and therefore likely to have a higher missing rate. Besides these two questions, missing data was low, suggesting that measures are largely acceptable to participants.

It is recommended that robust and rigorous assessment of an intervention’s therapeutic implications is conducted in an adequately sized definitive main trial [51]. However, within-group changes in all outcomes were in a positive direction. That there was a change in moderate to vigorous PA is especially encouraging given that our qualitative findings highlight a range of barriers to PA and surveys show that people reduce PA levels following stoma formation surgery [11–13]. Nevertheless, it is recommended that a decision to proceed to the main trial is based on clinical and patient judgement about a feasibility study’s findings rather than statistics [52]. Hence, we will continue to work with the Patient Advisory Group and enter discussions with organisations involving clinicians such as the UK Association of Stoma Care Nurses about the main trial. This will include a discussion about outcomes and measures included in this feasibility study and whether they remain relevant and justify patient burden. In particular, we will seek advice about the merits of including a measure of inflammation for IBD that involved blood samples.

### Intervention implementation

The number of PA consultations delivered by the instructors was used in this study as an objective measure of intervention fidelity. The median number of consultations was 8, which is 66% of what was planned. This is higher than other telephone-delivered PA intervention trials of similar duration [53, 54]. Nonetheless, the optimal number of consultations and duration (in weeks) required for a home-based PA programme to be effective is uncertain. The study suggests that it is useful to give participants a choice of method for a PA consultation since half were face-to-face and the other half were by VC or telephone. The latter methods are important because a key barrier to PA is travel [54]. Diaries show that participants completed 10 weeks (83%) of the 12-week prescribed PA programme, and 75% of prescribed exercises each week, suggesting that intervention adherence was high. Qualitative interviews suggest that instructors were using recommended behaviour change techniques [33] (monitoring, goal-setting, preferences, social support) as described in the intervention Manual and therefore were delivering the intervention as intended.

Qualitative interviews highlight the following barriers to being physically active in people with a stoma: fear of hernia, difficulties bending down, fatigue, pain, prolapse, surgical wounds, stoma appliance, and stigma. What is encouraging is that participants reported that the instructors helped them to address these barriers. Only one other study has explored deterrents for engaging in PA in people with a stoma. A qualitative study of 15 people with a stoma after surgery for rectal cancer found the following reasons for not engaging in PA: wounds from surgery, concerns about risk of hernia, fear of or actual pouch leaks, and feeling self-conscious, and the importance of having professionals able to address specific stoma-related concerns was also highlighted in a previous qualitative study [13]. That objectively measured PA increased post-intervention is further evidence suggesting that these stoma-related barriers to PA can be overcome in a structured PA programme for this patient group.

### Strengths and limitations

This is the first study of a manualised PA intervention for people with a stoma and therefore provides novel findings about the feasibility and acceptability of PA interventions for this patient group. There are a number of limitations. First, this study was conducted in one country in just three sites and it is important to recognise that contextual factors influence the success of complex intervention trials [55]. We therefore recommend a pilot study of the main trial in a larger number of sites prior to the main trial. Second, this study recruited only 30, mainly female people living with a stoma and we did not collect data about ethnicity. Hence, this is not representative sample and it could be argued that participants in this study do not represent the typical stoma population.

### Conclusions

The current PA intervention appears safe, feasible, and acceptable, and therefore, we recommend progression to a definitive main effectiveness trial subject to agreement from clinicians and patients. A pilot trial is advisable in recognition of contextual factors that may influence study procedures and trial implementation. It will be important that all sites commit to collecting important trial data to help interpret internal and external validity of a future main trial.

## Data Availability

The datasets used and/or analysed during the current study are available from the corresponding author on reasonable request.
